# Echocardiographic Evidence of Innate Aortopathy in the Human Intracranial Aneurysm

**DOI:** 10.1371/journal.pone.0100569

**Published:** 2014-06-25

**Authors:** Yong-Won Shin, Keun-Hwa Jung, Jeong-Min Kim, Young Dae Cho, Soon-Tae Lee, Kon Chu, Manho Kim, Sang Kun Lee, Moon Hee Han, Jae-Kyu Roh

**Affiliations:** 1 Department of Neurology, Laboratory for Neurotherapeutics, Biomedical Research Institute, Seoul National University Hospital, Seoul, South Korea; 2 Program in Neuroscience, Neuroscience Research Institute of SNUMRC, College of Medicine, Seoul National University, Seoul, South Korea; 3 Department of Neurology, Chung-Ang University Medical Center, College of Medicine, Chung-Ang University, Seoul, South Korea; 4 Department of Radiology, Seoul National University Hospital, Seoul, South Korea; 5 Department of Neurology, Armed Forces Capital Hospital, Seongnam, Gyeunggido, South Korea; INSERM U894, Centre de Psychiatrie et Neurosciences, Hopital Sainte-Anne and Université Paris 5, France

## Abstract

**Background:**

Intracranial aneurysm (IA) is significantly more prevalent in patients with coarctation of the aorta or bicuspid aortic valve than in the general population, suggesting a common pathophysiology connecting IA and aortopathy. Here, we analyzed echocardiographic aortic root dimension (ARD) in patients with IA to confirm this possibility.

**Methods:**

From January 2008 to December 2010, 260 consecutive patients with IA who were admitted to our institution for coil embolization or for acute stroke management and who also underwent echocardiography were enrolled. We hypothesized that patients with large, ruptured, or multiple IAs are more likely to harbor co-prevalent aortopathy as measured by ARD compared to patients with small, isolated, unruptured IAs. Eccentric group was defined as patients aged <55 years with at least one ruptured aneurysm, an aneurysm ≥7 mm in size, or multiple aneurysms; the remainder was classified into a non-eccentric group. Clinical, angiographic, and echocardiographic findings of the two groups were compared.

**Results:**

ARD was significantly larger in the eccentric group than in the non-eccentric group (*P* = 0.049), and the difference was confirmed by multivariable analysis (*P* = 0.02). Subgroup analysis of patients aged <55 years showed similar result for ARD (*P* = 0.03), whereas hypertension was more associated with the non-eccentric group (*P* = 0.01). In addition, height was inversely related to aneurysm size after adjustment for age, sex, weight, ARD, smoking status, and number of aneurysms (*P* = 0.004).

**Conclusions:**

A certain group of IA patients share a common intrinsic wall defect with aortopathy. Shared neural crest cell origin may give rise to this phenomenon.

## Introduction

Intracranial aneurysms (IAs), outpouches of weakened arterial walls, occur in 2–5% of the general population [1], [2]. IAs are usually asymptomatic, but rupture can have devastating consequences [3]. The pathophysiology of aneurysm formation, growth, and rupture is poorly understood. Local shear stress and inflammatory processes may explain vulnerable locations and the pathogenic process of IAs but have limited value for identifying potentially susceptible patients [4], [5]. Major risk factors such as hypertension, smoking, and female sex are insufficient to explain high interpersonal vulnerability differences [6]. Multiple aneurysms are found in 15–45% of patients with IA [7], [8], suggesting that a substantial number of patients are naturally aneurysm-prone. Epidemiological studies have shown that a developmental defect may be involved in the formation of multiple IAs [9], [10]. The contribution of innate arterial wall fragility to aneurysmal pathogenesis is supported by genetic, experimental, and clinical studies [9]–[21]. Therefore, characterization of intrinsic wall defects is important for improving our understanding of the nature of IAs.

Bicuspid aortic valve (BAV) and coarctation of the aorta (CoA) are congenital conditions that frequently occur with dilated aortic root and aortic or cervical dissection [11]. Intracranial aneurysms are also more prevalent in patients with BAV [12] or CoA [13], implying that IA and aortic pathologies share a common developmental defect. However, the rarity of patients presenting with these conditions makes it hard to extend the relationship to the general population. We postulated that a significant proportion of patients with IA have innate cerebral arteriopathy and aortopathy concomitantly, irrespective of BAV and CoA, and we designed the current study to uncover evidence of innate aortopathy in IA using aortic root dimension (ARD), a common echocardiographic marker.

## Methods

### Study population

Consecutive patients who were admitted to our institute between January 2008 and December 2010 for coil embolization for IA (hereafter “coiled patients,” *n* = 871) were initially screened. Of 871 coiled patients, 238 underwent transthoracic echocardiography. Indications for echocardiographic evaluation were not predefined owing to retrospective nature of this study, but in general, old age and/or cardiovascular risk factors underwent the echocardiography. For the same period, we also screened the existence of IA in patients who were admitted for acute ischemic stroke (hereafter “stroke patients,” *n* = 852). Stroke patients were selected because almost all patients underwent echocardiography, which is advantageous for getting echocardiographic data and mitigating selection bias in the retrospective study design. IAs were found in 59 stroke patients and all underwent transthoracic echocardiography during the same period. Three of 297 patients screened were excluded because of overlap between the two groups.

Patients with dissecting aneurysm (*n* = 9), fusiform aneurysm (*n* = 2), or extracranial carotid artery aneurysm (*n* = 1) were excluded because these aneurysm types have different pathologic characteristics. Patients with autosomal dominant polycystic kidney disease (*n* = 7), Ehler-Danlos syndrome type IV (*n* = 1), or granulomatous arteritis with multiple aneurysms (*n* = 1) were excluded because these conditions are associated with aneurysm formation. Patients with missing height information (*n* = 13) were excluded because height is a principal determinant of ARD [22]. A total of 260 patients were finally included in the analysis.

Clinical information about the coiled patients was retrieved from the database prospectively maintained by trained interventionists using a standardized case registration form. Data from stroke patients were collected from the nationwide Korean Stroke Registry, which was started in 1999 to investigate the characteristics, mechanisms, risk factors, and neurological outcomes of stroke in Korea [23]. The stroke registry is based on a predefined registration form filled out by trained physicians or research nurses, and data fidelity was confirmed by experienced vascular neurologists. The study was approved by the Human Subject Research Committee of the Seoul National University Hospital. Written informed consent was obtained from all patients or their next of kin.

### Data acquisition and interpretation

The following clinical information was collected from each patient at admission: demographic characteristics (age at admission, sex, height, and weight), atherosclerotic risk factors (hypertension [HTN], diabetes mellitus [DM], hyperlipidemia, and smoking status), and history of atrial fibrillation, coronary artery disease (CAD; angina pectoris or myocardial infarction), or cerebrovascular accident (stroke or transient ischemic attack). HTN was identified when the blood pressure was repeatedly measured at >140 mm Hg systolic or >90 mm Hg diastolic or when the patients were on antihypertensive medication. Hyperlipidemia was defined as an elevated LDL cholesterol (≥160 mg/dl) or total cholesterol (≥240 mg/dl) or treatment with a lipid-lowering agent. DM diagnosis was based on a previous history of DM or the diagnostic guidelines of the American Diabetes Association [24]. Body mass index was calculated as weight/(height)^2^ (kg/m^2^), and body surface area (BSA) was calculated with the Mosteller formula [25]. We also searched medical records and available computed tomography data to determine whether patients had CoA.

Echocardiographic data included ARD, left atrial (LA) dimension, interventricular septal end diastolic dimension (IVSd), left ventricular end diastolic posterior wall dimension (LVPWd), and presence of aortic valve pathology such as aortic regurgitation, aortic stenosis, or BAV. Given that ARD is frequently associated with aortic pathology such as ascending aortic aneurysm, dissection, and coarctation of aorta [11], ARD was selected as a potential marker for aortopathy. Other routine markers, LA dimension, IVSd, and LVPWd were selected for comparisons. ARD was measured by the leading-edge-to-leading-edge technique, at end diastole or at the widest dimension best visualized regardless of the cardiac cycle [26]. LA dimension, LVPWd, and IVSd were also measured according to the recommendations of the American Society of Echocardiography [26]. All echocardiographic findings were double checked by two experienced cardiologists at the time of evaluation.

IAs were diagnosed and characterized on the basis of conventional angiography or magnetic resonance angiography. Maximal diameter of IA was determined, and the largest aneurysm was used in the analysis if the patient had multiple aneurysms. Aneurysms of ≥7 mm were considered large according to previous studies [27], [28]. All imaging studies were evaluated by two experienced neuroradiologists.

### Statistical analysis

Patient demographics and clinical features were analyzed using descriptive statistics. Data are expressed as means ± standard deviations or numbers (percentages). Echocardiographic markers were analyzed as absolute measures or as indexed to either BSA or height. For comparisons between two groups, the Pearson χ^2^ test or the Fisher two-tailed exact test was used for categorical variables, and the Student *t* test or the Mann-Whitney U test was used for continuous variables. One-way ANOVA was used for multiple comparisons. Bivariate correlations were performed with Pearson coefficients. Variables with clinical importance or with *P* values of <0.10 in univariable analysis were included in multivariable analysis. For subgroup analysis of patients aged <55, we performed backward stepwise logistic regression analysis with a *P* value for entry of 0.05 and a *P* value for removal of 0.1. For multiple linear regression analysis, independent variables were selected according to the result of correlation analysis. Statistical significance was set at *P*<0.05, and SPSS (ver. 18.0 SPSS Inc., Chicago, IL) was used for the analyses.

## Results

### Age distribution of study population

**Figure 1:**
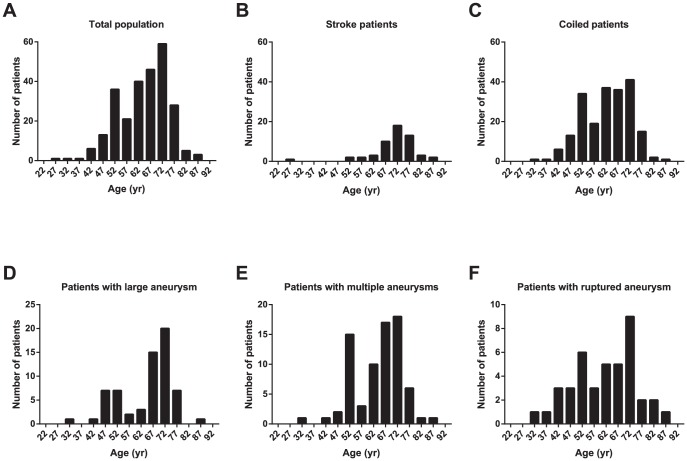
Age distribution of the study population. (a) Age distribution of the total population (*n* = 260) was bimodal; there were a large number of patients aged 50–54 years. (b) The stroke patients (*n* = 57) were relatively old, and the distribution was unimodal. (c) Age distribution of the coiled patients (*n* = 206) demonstrate another peak which contributed to the bimodality of the total population. Patients with (d) large IAs (≥7, *n* = 64), (e) multiple aneurysms (*n* = 75), and (f) a ruptured aneurysm (*n* = 41) also showed bimodal age distributions. The labels on the *x*-axis indicate mean ages of 5-year age groups starting at age 20.

The age distribution of the study group showed heterogeneity but skewed towards older ages (64.1±10.3 years, Figure 1A). Stroke patients with IA consisted mainly of an old age population (70.4±9.1 years, Figure 1B), whereas coiled patients had an additional population with younger ages (Figure 1C). The age distribution of patients with IAs with eccentric characteristics (large, multiple, and rupture) were bimodal (Figure. 1D–F).

### Characteristics of eccentric and non-eccentric groups

To separate patients with potential intrinsic arterial wall defects, we selected an eccentric group consisting of patients aged <55 years with at least one of the following features: aneurysm size ≥7 mm, multiple aneurysms, or rupture presentation; all other patients were categorized as non-eccentric. As owing to the group definitions, age and the number of aneurysms and ruptured aneurysms differed between the groups (Table S1). Aneurysms tended to be larger in the eccentric group with a borderline significance. The non-eccentric group was shorter than the eccentric group, perhaps because of age differences. Cerebrovascular risk factors (HTN, DM, and hyperlipidemia) and history of stroke or CAD were significantly higher in the non-eccentric group than in the eccentric group. Absolute values of ARD of the eccentric group was significantly larger than that of the non-eccentric group. The ARDs indexed to BSA or to height showed no significant differences. LA dimension was significantly smaller in eccentric group when indexed to BSA and height but not as absolute measures. IVSd and LVPWd were not significantly different between the groups. BAV and CoA were not found in the study population.

### Independent association of ARD with eccentricity

Multivariable analysis showed that only ARD and age differed significantly between the two groups (Table 1). Female sex was included in the model because of its significant effect on aneurysm characteristics [6], but it was not significant after adjustment. Atherosclerotic risk factors (HTN, DM, hyperlipidemia, smoking status, and history of stroke or CAD) were not associated with eccentricity. LA dimension did not differ between the groups. Additional logistic regression analysis with other echocardiographic findings (e.g., IVSd and LVPWd) also showed no difference between the groups (Table S2). The results were not different when only the coiled patients were analyzed (Table S2 and S3). All of the results were reconfirmed by the analyses using indexed echocardiographic measures. Because of the significant age discrepancy between the two groups, we also performed subgroup analysis with only patients who were aged <55 years (eccentric group, *n* = 33; non-eccentric group, *n* = 25). Univariable analysis showed no significant difference in age between the groups (*P* = 0.61). Atherosclerotic risk factors and echocardiographic markers were also not different between the groups except for HTN (*P* = 0.03). Logistic regression analysis by a backward stepwise selection method selected three variables in the final model: HTN, ARD, and height (Table 2); ARD remained significantly different, while age differences were eliminated. HTN was more frequently associated with non-eccentricity, suggesting that some mechanism other than hypertension is implicated in eccentricity. Patients in the eccentric group tended to be short, although the statistical significance was marginal. Analyses using indexed values also showed similar trend except for height which did not show independent relationship with eccentricity.

**Table 1:** Results of multivariable logistic regression analysis of determinants of eccentricity.

	Using absolute echocardiographic measurement		Indexed to BSA		Indexed to height	
	OR (95% CI)	*P* value	OR (95% CI)	*P* value	OR (95% CI)	*P* value
Age	0.78 (0.70–0.86)	<0.001	0.82 (0.76–0.88)	<0.001	0.81 (0.75–0.87)	<0.001
Female	1.87 (0.29–12.10)	0.51	1.27 (0.23–6.98)	0.78	1.38 (0.25–7.60)	0.71
Height	0.98 (0.90–1.07)	0.62	1.01 (0.93–1.10)	0.83	1.00 (0.93–1.08)	0.96
ARD	1.19 (1.03–1.36)	0.02	1.22 (1.00–1.49)	0.048	1.26 (1.04–1.53)	0.02
LA dimension	0.99 (0.91–1.07)	0.75	0.98 (0.86–1.12)	0.78	1.00 (0.88–1.12)	0.94
Hypertension	0.40 (0.12–1.37)	0.15	0.44 (0.14–1.34)	0.15	0.40 (0.13–1.24)	0.11
Diabetes mellitus	0.70 (0.07–7.23)	0.77	0.60 (0.06–5.69)	0.66	0.61 (0.06–5.86)	0.67
Hyperlipidemia	0.44 (0.10–2.02)	0.29	0.51 (0.13–2.05)	0.34	0.48 (0.12–1.95)	0.30
Former or current smoking	1.97 (0.34–11.46)	0.45	1.65 (0.33–8.26)	0.54	1.90 (0.39–9.28)	0.43
History of stroke or CAD*	0.34 (0.05–2.29)	0.27	0.41 (0.07–2.37)	0.32	0.38 (0.06–2.32)	0.29

*Patients with ischemic stroke, transient ischemic attack, or coronary artery disease (angina pectoris or myocardial infarction) were included.

BSA: body surface area; OR: odds ratio; CI: confidence interval; CAD: coronary artery disease; ARD: aortic root dimension; LA: left atrial.

**Table 2:** Subgroup analysis of patients aged <55 years.

	Using absolute echocardiographic measurement		Indexed to BSA		Indexed to height	
	OR (95% CI)	*P* value	OR (95% CI)	*P* value	OR (95% CI)	*P* value
Hypertension	0.16 (0.04–0.58)	0.005	0.162 (0.04–0.60)	0.006	0.18 (0.05–0.62)	0.007
ARD	1.18 (1.02–1.37)	0.03	1.39 (1.05–1.85)	0.02	1.31 (1.03–1.66)	0.03
Height	0.93 (0.86–1.00)	0.06	-		-	

OR: odds ratio; CI: confidence interval; ARD: aortic root dimension.

### Relationship between height and other variables

**Figure 2:**
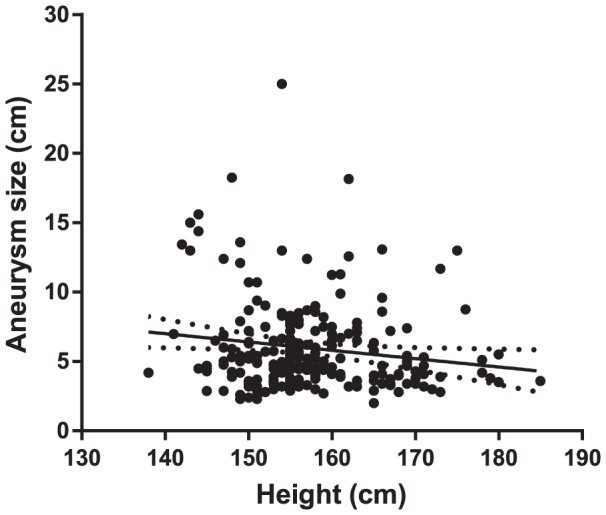
Scatter plot of aneurysm size versus height. Aneurysm size tended to decrease with increasing height. The fitted regression line for the association is shown as a solid line with the 95% confidence intervals shown as dotted lines (*R* ^2^ = 0.046).

The initial unadjusted data indicated that patients in the eccentric group were taller than those in the non-eccentric group, but the difference was not significant after adjustment for age and other factors. The relationship between height and eccentricity was reversed when the analysis was restricted to patients aged <55 years, however this relationship was not statistically significant and no relationship was found when using indexed data. Owing to the unclear relationship between height and eccentricity, further analysis to find an association between height and IA was conducted. Correlation analysis showed that age, sex, weight, ARD, smoking status, and size and number of aneurysms were correlated with height (*P*≤0.001, except for the number of aneurysms, *P* = 0.003). Linear regression with all these variables showed that age, sex, weight, and aneurysm size were independently associated with height (Table 3). Height and aneurysm size were negatively correlated, and patients with a large aneurysm tended to congregate in the low-height group (Figure 2).

**Table 3:** Results of multiple linear regression analysis of association between height and other variables.

	β± SE	*P* value
Age	−0.10±0.03	0.002
Female	−10.83±0.79	<0.001
Weight	0.21±0.04	<0.001
Aneurysm size	−0.29±0.10	0.004

Stepwise selection method was used for multiple regression analysis. Body surface area and body mass index were excluded from the model owing to their multicollinearity with weight.

*β*: non-standardized regression coefficient; SE: standard error.

## Discussion

In this study, we tried to identify a pathological interrelationship between intracranial arteries and the ascending aorta in patients with IA. The tunica media of both arteries have a common embryological origin (neural crest cells) and a similar structure of cross-linked elastin and collagen [4], [11], [29]–[32], but data supporting a pathological relationship between diseases involving the two structures is limited [12], [13]. Moreover, these studies involved patients with rare disease conditions. Some genetic variation associated with both IA and aortic aneurysm has been reported [19], but investigations under routine clinical practice are lacking. Here, we assessed for a pathogenic link between the two diseases by evaluating ARD without restriction to specific disorders.

The age distribution of the study population was bimodal. Although old age and known risk factors for IA may have some contribution to the older-age peak, the younger-age peak suggests that other mechanisms operate. Intrinsic arterial wall defects could affect aneurysm formation, growth, and rupture, leading to early detection. However, there are several factors that affect the age distribution. Patients with multiple risk factors at middle and advanced age tend to have further diagnostic evaluation including angiography. Moreover, patients who underwent screening brain imaging in midlife might contribute to the bimodal age distribution, considering that most IAs are asymptomatic. The markedly high prevalence of IA among patients aged 50–54 years in our data supports this possibility. However, this age distribution likely indicates that a significant number of IAs form before age 55 or before vascular risk factors sufficiently affect IA generation.

ARD tends to increase with age and height [22]. However, we found that the ARD in the eccentric group was significantly larger than that in the non-eccentric group, despite the younger age of the former. Subgroup analysis intended to eliminate the effect of the age distribution difference reconfirm this difference. The large ARD in the eccentric group may have been affected by height; however, multivariable analysis confirmed that the association between large ARD and eccentricity was independent of height, and subgroup analysis with absolute values of echocardiographic markers, but not with indexed values, even showed a trend toward short stature in the eccentric group. Furthermore, HTN, known to be associated with an increase in ARD, was more related to non-eccentricity in the subgroup analysis. The difference was independent of aortic valve pathology (BAV, aortic stenosis and aortic regurgitation).

A previous study by Curtis et al. [33] showed that fewer patients with both CoA and IA had BAV or ascending aortopathy than patients with CoA and without IA. However, the difference was not statistically significant (*P* = 0.36), was not adjusted for other factors, and have different demographics. Another study by Schievink et al. [12] found no relationship between thoracic aortic dilatation and the presence of IA in a bicuspid valve group. But only 6 of patients with BAV had IA in this subgroup analysis. More to the point, we focused on the eccentricity, not the presence, of IA and its relationship with ARD. The abovementioned studies only consider the presence of IA and do not specifically consider the nature of IA in their analysis. Considering the pathophysiological heterogeneity in IA, we hypothesized that a certain group, not all, of IA patients have concomitant aortopathy and defined the eccentric group as accordingly.

A plausible explanation for the link between IA and ascending aortic pathology is neurocristopathy arising from the embryonic neural crest. The neural crest is a key structure in the vertebrate embryo and plays a major role in the development of head structures, the aortic arch and cervicocephalic arteries, skin melanocytes, much of the peripheral nervous system, some endocrine cells, and various other structures [29]–[32]. Combinations of BAV, CoA, aortic root dilatation, aortic and cervical arterial dissection, and histologically, cystic medial wall degeneration are considered as phenotypes of neurocristopathy because the aortic valve, the medial wall of the aortic arch, and the cervicocephalic arteries derive from neural crest cells [11], [29]–[31], and abnormal development of these cells can result in vascular fragility [31]. Neural crest cells make smooth muscle cells and pericytes in the medial wall of intracranial arteries [32]. Phenotypic modulations of vascular smooth cells in the medial layer disrupt the structural integrity of the vessel walls and contribute to IA formation [4]. There are also anecdotal reports of an association between IA and Parry-Romberg syndrome [14], [15] and congenital heart diseases [16], suggesting neurocristopathy as a common factor. A higher detection rate of IA in neurofibromatosis type 1, a neurocristopathy spectrum disease, was reported in a case-control study [17]. Southerland et al. recently suggested that the association between IA, Moyamoya disease, fibromuscular dysplasia, and cervical dissection, which share common pathological features of transformation of smooth muscle cells and degraded elastic laminae [18], is reminiscent of neurocristopathy. These studies, taken together with our findings on the relationship between IA and ARD, imply that some intrinsic intracranial arterial wall defects may also affect the ascending aorta, and, conclusively, could be regarded as one part of neurocristopathy.

Other echocardiographic values analyzed in our study support this perspective. During cardiogenesis, neural crest cells play a role in aorticopulmonary septation and aortic valve and left ventricular outflow tract development [29]–[31] but are apparently not involved in the formation of the atrial chambers and most of the ventricular wall structures, which are derived mainly from cardiac mesenchyme [30]. Unlike ARD, LVPWd, IVSd, and LA dimension did not differ significantly between the two groups. The differential contributions of these echocardiographic measurements to eccentricity support the different embryological origins of these structures.

Recent genetic evidence supports the implication of neurocristopathy in IA. A genome-wide association study identified several loci variants associated with IA; specifically, single-nucleotide polymorphisms in chromosome 9p21.3 are associated with various arterial disorders including CAD and aortic aneurysm [19]. Underexpression of ANRIL, a large noncoding RNA in the 9p21.3 locus, seems to mediate neural crest-derived tumors including melanomas and plexiform neurofibroma [34]–[36] along with IA and CAD [19], [34]–[36]. A recent ablation study that revealed the heterogeneous origin of coronary artery smooth muscle cells also investigated the role of endothelin receptor type-A (EDNRA) in craniofacial and cardiovascular development from neural crest cells [37]. An association between IA and single-nucleotide polymorphisms in or near the EDNRA gene [19]–[21] also supports our concept. Taken together, the results of genetic, embryological, and clinical studies suggest that neurocristopathy is a unifying pathogenesis of these seemingly unrelated disorders, but additional studies are required to solidify the involvement of neural crest in the disorders.

In our study, patients with large aneurysms tended to be short, which provides additional evidence of systemic or developmental involvement in IA. Neural crest cells differentiate into bone and cartilage and into thyroid parafollicular cells, which secrete calcitonin, and can affect the development of the adenohypophysis, which produces growth-related hormones [29], [38]. Neurofibromatosis type 1 is a representative disorder in which neurocristopathy can accompany short stature and osteoporosis [39]. However, in our study, the height difference between the eccentric and non-eccentric groups was not statistically significant, and other developmental and systemic diseases may affect both IA and height. Further investigation is needed to explain this phenomenon.

Cardiovascular risk factors were more prevalent in our cohort than in the general population, owing to retrospective inclusion of subjects with available echocardiography data. Known racial/ethnic differences in IA phenotype also limit generalization of our result to other ethnic populations. The possibility of undiagnosed monogenic connective tissue diseases should be considered as well because these were retrospectively excluded from the analysis. Lastly, the small sample size and the use of only one marker, ARD, for aortopathy are another limitation of this study. A population-based study and the use of other markers of aortic pathology would be needed to validate the findings.

ARD, a novel indicator for a shared pathogenesis of IA and aortopathy, and embryological implication as neurocristopathy sheds a new light on future work to understand the pathophysiology of IAs. Additional intrinsic properties of IA should be characterized for better diagnosis and treatment of IA.

## Supporting Information

Table S1 Demographic and clinical features of the study population.
(DOCX)

Table S2 P-values for other echocardiographic markers.
(DOCX)

Table S3 Results of multivariable logistic regression analysis of determinants of eccentricity in coiled patients.
(DOCX)
