# Supplementary material for: Echocardiographic Evidence of Innate Aortopathy in the Human Intracranial Aneurysm
Source: PLoS One. 2014 Jun 25;9(6):e100569. doi: 10.1371/journal.pone.0100569 (PMC4070985; doi:10.1371/journal.pone.0100569)
Supplement: Table S1 — Demographic and clinical features of the study population. (DOCX) [file pone.0100569.s001.docx]

**Table S1. Demographic and clinical features of the study population.**

|  | Total (*n* = 260) | Eccentric group (*n* = 33) | Non-eccentric group (*n* = 227) | *P* value |
| --- | --- | --- | --- | --- |
| Age, years | 64.1 ± 10.3 | 49.2 ± 5.2 | 66.2 ± 9.0 | <0.001 |
| Female | 180 (69.2) | 21 (63.6) | 159 (70.0) | 0.46 |
| Hypertension | 190 (73.1) | 13 (39.4) | 177 (78.0) | <0.001 |
| Diabetes mellitus | 52 (20.0) | 1 (3.0) | 51 (22.5) | 0.009 |
| Hyperlipidemia | 76 (29.2) | 4 (12.1) | 72 (31.7) | 0.02 |
| Former or current smoking | 50 (19.2) | 10 (30.3) | 40 (17.6) | 0.08 |
| History of stroke or CAD^*^ | 60 (23.1) | 2 (6.1) | 58 (25.6) | 0.01 |
| Atrial fibrillation | 14 (5.4) | 0 (0.0) | 14 (6.2) | 0.23 |
| Height, cm | 157.8 ± 8.5 | 160.4 ± 6.8 | 157.4 ± 8.6 | 0.06 |
| Weight, kg | 60.0 ± 10.5 | 61.2 ± 10.0 | 59.8 ± 10.6 | 0.46 |
| Body mass index, kg/m^2^ | 24.0 ± 3.5 | 23.8 ± 3.5 | 24.1 ± 3.5 | 0.62 |
| Body surface area, m^2^ | 1.62 ± 0.17 | 1.65 ± 0.15 | 1.61 ± 0.17 | 0.27 |
| Echocardiography |  |  |  |  |
| IVSd (absolute), mm | 9.1 ± 2.2 | 8.8 ± 1.6 | 9.1 ± 2.3 | 0.52 |
| (indexed to BSA), mm/m^2^ | 5.7 ± 1.4 | 5.4 ± 1.1 | 5.7 ± 1.5 | 0.24 |
| (indexed to height), mm/m | 5.8 ±1.4 | 5.5 ± 1.0 | 5.8 ± 1.5 | 0.22 |
| LVPWd (absolute), mm | 8.9 ± 2.2 | 9.3 ± 2.0 | 8.9 ± 2.3 | 0.29 |
| (indexed to BSA), mm/m^2^ | 5.6 ±1.4 | 5.7 ± 1.4 | 5.6 ± 1.5 | 0.68 |
| (indexed to height), mm/m | 5.7 ± 1.4 | 5.8 ± 1.3 | 5.7 ±1.5 | 0.73 |
| LA dimension (absolute), mm | 39.5 ± 7.7 | 37.1 ± 6.9 | 39.8 ± 7.7 | 0.06 |
| (indexed to BSA), mm/m^2^ | 24.5 ± 4.8 | 22.7 ±5.0 | 24.8 ± 4.8 | 0.01 |
| (indexed to height), mm/m | 25.0 ± 4.8 | 23.0 ± 4.6 | 25.4 ± 4.8 | 0.01 |
| ARD (absolute), mm | 31.8 ± 4.5 | 33.3 ± 5.9 | 31.6 ± 4.2 | 0.049 |
| (indexed to BSA), mm/m^2^ | 19.8 ± 2.9 | 20.4 ± 3.9 | 19.7 ± 2.7 | 0.22 |
| (indexed to height), mm/m | 20.2 ± 2.7 | 20.7 ± 3.6 | 20.1 ± 2.6 | 0.35 |
| Aortic stenosis | 6 (2.3) | 0 (0.0) | 6 (2.6) | 1.00 |
| Aortic regurgitation | 41 (15.8) | 3 (9.1) | 38 (16.7) | 0.26 |
| Characteristics of aneurysm |  |  |  |  |
| Size, mm | 5.5 ± 3.4 | 6.6 ± 3.2 | 5.4 ± 3.4 | 0.051 |
| Number | 1.4 ± 0.8 | 2.0 ± 1.0 | 1.4 ± 0.7 | 0.003 |
| Rupture | 41 (15.8) | 14 (42.4) | 27 (11.9) | <0.001 |
| Location |  |  |  | 0.78 |
| Anterior cerebral artery | 9 (3.5) | 1 (3.0) | 8 (3.5) |  |
| Anterior communicating artery | 68 (26.2) | 9 (27.3) | 59 (26.0) |  |
| Internal carotid artery | 77 (29.6) | 13 (39.4) | 64 (28.2) |  |
| Middle cerebral artery | 36 (13.8) | 4 (12.1) | 32 (14.1) |  |
| Posterior communicating artery | 40 (15.4) | 3 (9.1) | 37 (16.3) |  |
| Posterior circulation | 30 (11.5) | 3 (9.1) | 27 (11.9) |  |

Data are presented as means ± standard deviations or numbers (percentages).

^*^Patients with ischemic stroke, transient ischemic attack, or coronary artery disease (angina pectoris or myocardial infarction) were included.

CAD: coronary artery disease; IVSd: interventricular septal end diastolic dimension; LVPWd: left ventricular end diastolic posterior wall dimension; LA: left atrial; ARD: aortic root dimension.
