# Supplementary material for: Echocardiographic Evidence of Innate Aortopathy in the Human Intracranial Aneurysm
Source: PLoS One. 2014 Jun 25;9(6):e100569. doi: 10.1371/journal.pone.0100569 (PMC4070985; doi:10.1371/journal.pone.0100569)
Supplement: Table S2 — P-values for other echocardiographic markers. (DOCX) [file pone.0100569.s002.docx]

**Table S2. P-values for other echocardiographic markers.**

|  | Total subjects | | | Coiled patients | | |
| --- | --- | --- | --- | --- | --- | --- |
|  | Absolute value | Indexed to BSA | Indexed to height | Absolute value | Indexed to BSA | Indexed to height |
| IVSd | 0.69 | 0.77 | 0.34 | 0.35 | 0.80 | 0.35 |
| LVPWd | 0.35 | 0.46 | 0.21 | 0.21 | 0.48 | 0.21 |

Differences of each echocardiographic marker between the eccentric and non-eccentric groups were evaluated using multivariable analysis adjusting for age, sex, height, aortic root dimension (absolute or indexed values as appropriate), left atrial dimension (absolute or indexed values as appropriate), hypertension, diabetes mellitus, hyperlipidemia, former or current smoking, and history of stroke or coronary artery disease. P-values for each analysis are displayed.

BSA: body surface area; IVSd: interventricular septal end diastolic dimension; LVPWd: left ventricular end diastolic posterior wall dimension.
