# Supplementary material for: Echocardiographic Evidence of Innate Aortopathy in the Human Intracranial Aneurysm
Source: PLoS One. 2014 Jun 25;9(6):e100569. doi: 10.1371/journal.pone.0100569 (PMC4070985; doi:10.1371/journal.pone.0100569)
Supplement: Table S3 — Results of multivariable logistic regression analysis of determinants of eccentricity in coiled patients. (DOCX) [file pone.0100569.s003.docx]

**Table S3. Results of multivariable logistic regression analysis of determinants of eccentricity in coiled patients.**

|  | Using absolute echocardiographic measurement | | Indexed to BSA | | Indexed to height | |
| --- | --- | --- | --- | --- | --- | --- |
|  | OR (95% CI) | *P* value | OR (95% CI) | *P* value | OR (95% CI) | *P* value |
| Age | 0.78 (0.70–0.86) | <0.001 | 0.79 (0.71–0.87) | <0.001 | 0.78 (0.70–0.86) | <0.001 |
| Female | 1.87 (0.29–2.10) | 0.51 | 1.78 (0.28–1.50) | 0.54 | 1.86 (0.29–2.02) | 0.51 |
| Height | 0.98 (0.90–1.07) | 0.62 | 1.02 (0.93–1.11) | 0.70 | 1.01 (0.93–1.10) | 0.84 |
| ARD | 1.19 (1.03–1.36) | 0.02 | 1.26 (1.01–1.58) | 0.04 | 1.30 (1.05–1.62) | 0.02 |
| LA dimension | 0.99 (0.91–1.07) | 0.75 | 0.97 (0.84–1.12) | 0.70 | 0.98 (0.86–1.12) | 0.80 |
| Hypertension | 0.40 (0.12–1.37) | 0.15 | 0.44 (0.13–1.50) | 0.19 | 0.41 (0.12–1.38) | 0.15 |
| Diabetes mellitus | 0.70 (0.07–7.23) | 0.77 | 0.68 (0.07–6.84) | 0.74 | 0.69 (0.07–7.09) | 0.75 |
| Hyperlipidemia | 0.44 (0.10–2.02) | 0.29 | 0.51 (0.12–2.22) | 0.37 | 0.45 (0.10–2.05) | 0.30 |
| Former or current smoking | 1.97 (0.34–1.46) | 0.45 | 1.84 (0.31–1.01) | 0.51 | 2.04 (0.35–1.86) | 0.42 |
| History of stroke or CAD^*^ | 0.34 (0.05–2.29) | 0.27 | 0.38 (0.06–2.35) | 0.30 | 0.34 (0.05–2.30) | 0.27 |

^*^Patients with ischemic stroke, transient ischemic attack, or coronary artery disease (angina pectoris or myocardial infarction) were included.

BSA: body surface area; OR: odds ratio; CI: confidence interval; ARD: aortic root dimension; LA: left atrial.
